# Prognostic implication of morphology, cyclinE2 and proliferation in EBV-associated T/NK lymphoproliferative disease in non-immunocompromised hosts

**DOI:** 10.1186/s13023-014-0165-x

**Published:** 2014-12-05

**Authors:** Siok-Bian Ng, Koichi Ohshima, Viknesvaran Selvarajan, Gaofeng Huang, Shoa-Nian Choo, Hiroaki Miyoshi, Shi Wang, Hsin-Chieh Chua, Allen Eng-Juh Yeoh, Thuan-Chong Quah, Liang-Piu Koh, Poh-Lin Tan, Wee-Joo Chng

**Affiliations:** Department of Pathology, National University Cancer Institute of Singapore, National University Health System, Yong Loo Lin School of Medicine, Cancer Science Institute of Singapore, National University of Singapore, Singapore, Singapore; Department of Pathology, Kurume University, Asahimati 67, Kurume, 830-0011 Japan; Department of Pathology, National University Health System, Singapore, Singapore; Cancer Science Institute of Singapore, National University of Singapore, Singapore, Singapore; Department of Paediatrics, National University Health System, Singapore, Singapore; Department of Haematology-Oncology, National University Health System, Singapore, Singapore; Department of Haematology-Oncology, National University Cancer Institute of Singapore, National University Health System, Yong Loo Lin School of Medicine, Cancer Science Institute of Singapore, 1E, Kent Ridge Rd, Singapore, 119228 Singapore; Department of Pathology, National University Hospital, 5 Lower Kent Ridge Road, Main Building, Level 3, Singapore, 119074 Singapore

**Keywords:** EBV-associated T/NK lymphoproliferative disease, Molecular signature, Morphology, Cyclin E2, Proliferation, Prognosis

## Abstract

**Background:**

EBV-associated T/NK-cell lymphoproliferative diseases (TNKLPD) is a rare spectrum of disease that occurs more commonly in Asia, and Central and South America. It commonly affects children and young adults and is an aggressive disease that is poorly understood with no known biologic markers that can predict prognosis. The systemic form of TNKLPD includes chronic active EBV infection of T/NK type, aggressive NK cell leukemia and systemic EBV + T-cell lymphoproliferative disease of childhood.

**Methods:**

In this study, we analyse the clinicopathologic and genetic features of 22 cases of systemic TNKLPD in non-immunocompromised patients, including chronic active EBV infection of T/NK cell type and systemic EBV + T-cell lymphoproliferative disease of childhood. We also performed gene expression profiling in a subset of cases to identify markers that may be of prognostic relevance and validated our results using immunohistochemistry.

**Results:**

The median age is 14.9 years and two of our 22 cases occurring in patients older than 30 years. Fifteen of 17 cases (88%) with adequate data were of T-cell origin. Eleven of 22 cases revealed polymorphic cellular infiltrate (P-group) while the rest showed monomorphic lymphoid infiltrate (M-group). We found a significant difference in survival between P-group vs M-group patients with median survival not yet reached in P-group, and 1 month in M-group (p = 0.0001), suggesting a role for morphology in predicting patient outcome. We also performed gene expression profiling in a subset of patients and compared the genes differentially expressed between P-group and M-group cases to identify markers of prognostic value. We identified cyclin E2 gene and protein to be differentially expressed between patients with good outcome (P-group, median expression 8%) and poor outcome (M-group, median expression 42%) (p = 0.0005). In addition, the upregulation of cyclin E2 protein in M-group cases correlated with a higher Ki67 proliferation rate (Pearson correlation r = 0.73, p = 0.0006) detected by immunohistochemistry. High cyclin E2 expression was also significantly associated with shorter survival (p = 0.0002).

**Conclusion:**

Our data suggests the potential role of monomorphic morphology, high cyclin E2 and Ki67 expression as adverse prognostic factors for TNKLPD.

**Electronic supplementary material:**

The online version of this article (doi:10.1186/s13023-014-0165-x) contains supplementary material, which is available to authorized users.

## Background

The term EBV-associated T/NK-cell lymphoproliferative disorders (TNKLPD) encompasses a spectrum of disease entities in non-immunocompromised patients characterised by an Epstein-Barr virus (EBV)-infected, cytotoxic T or NK-cell proliferation that is polyclonal or monoclonal. The systemic form of TNKLPD includes chronic active EBV infection of T/NK-cell type (CAEBV), aggressive NK cell leukemia (ANKL) and systemic EBV-positive T-cell lymphoproliferative disease of childhood (STLPDC) [1-4]. TNKLPD is rare in the Western population and occurs with increased frequency in Asians, Native Americans from Central and South America and Mexico [1,2] Morphologically, it is characterized by a wide-ranging cytological appearance from reactive appearance to overt leukemia/lymphoma [5-7]. The diagnosis of TNKLPD can be challenging because cytologic features may be deceptively benign despite the frequently aggressive clinical behaviour.

Historically, the terminology and classification of TNKLPD have been problematic as this process has been described under a variety of terms including: fulminant EBV + T-cell lymphoproliferative disease (LPD) of childhood [8], fatal infectious mononucleosis; fulminant hemophagocytic syndrome in children in Taiwan [9], fatal EBV-associated hemophagocytic syndrome in Japan [10] and severe CAEBV [11]. However, recent efforts have been made to achieve international consensus on the nomenclature and classification of TNKLPD and it is now recognized that most of the EBV-associated T/NK LPD in immunocompetent children and young adults with systemic presentation belong to the same spectrum of disease, including CAEBV and STLPDC. A consensus meeting was held at the National Institute of Health (NIH) in 2008 and it was recommended that the term CAEBV should be applied to systemic LPDs that are not frank lymphomas and that arise during primary infection and persist for over 6 months. On the other hand, the term ‘systemic EBV-positive T-cell LPD’, as adopted by the WHO classification, is the preferred pathologic designation over CAEBV for those cases that are clonal with an aggressive clinical course and require aggressive treatment [12]. A clinicopathological categorization of TNKLPD was proposed by Ohshima et al. in 2008 [13]. According to the proposal, EBV-associated T/NK LPD in children can be categorised into 4 groups (A1, A2, A3 and B) based on morphology of the infiltrate, clonality and clinical presentation. A consensus report following the 4th Asian Hematopathology Workshop on the classification and terminology of TNKLPD was also recently published [14]. In this report, TNKLPD can be divided into systemic and cutaneous forms (see Additional file 1, for a summary of the proposed classifications of TNKLPD). The systemic form includes CAEBV-T/NK, ANKL and STLPDC.

In this study, we analysed the clinicopathologic and genetic features of 22 cases of systemic form of TNKLPD in Singapore and Japan using the criteria recommended by WHO and NIH in 2008, including CAEBV and STLPDC. We found that patients with polymorphic infiltrate (P-group) have a significantly better outcome than cases with monomorphic infiltrate (M-group). We then performed, for the first time, gene expression profiling (GEP) in a subset of patients and compared the gene signature between P-group and M-group patients with good and poor outcome, respectively, in order to identifying targets that may be important in predicting outcome.

## Methods

### Case selection and definition

Patients with a diagnosis of EBV-associated T/NK-cell lymphoproliferative disorder without known underlying immune deficiency were identified from the archives of the Department of Pathology, National University Hospital (NUH), and Kurume University, from 2003 to 2013. Cases were reviewed, additional immunohistochemistry and in-situ hybridization for EBV-encoded small RNA (EBER) were performed and the cases were classified according to the 2008 WHO lymphoma classification and nomenclature proposed following the NIH consensus report in 2008 (Table 1) [12]. The determination of T vs NK cell lineage is based on the TCRG gene clonality and protein expression of TCRαβ and TCRγδ. Extranodal nasal-type NK/T-cell lymphoma cases were excluded. A total of 22 cases with adequate material for workup and fulfilled the diagnostic criteria were identified and included CAEBV of T/NK type, and systemic EBV-associated T-cell LPD of childhood. The clinical history, previous biopsies and laboratory results were reviewed for evidence of hemophagocytic lymphohistiocytosis (HLH). Hemophagocytic lymphohistiocytosis (HLH) is defined by at least five of the eight criteria including fever, splenomegaly, bicytopenia, hypertriglyceridemia, and/or hypofibrinogenemia, hemophagocytosis, low/absent NK-cell-activity, hyperferritinemia, and high-soluble interleukin-2-receptor levels [15].

**Table 1:** **Pathologic and genetic features and classification of EBV + T/NK-cell lymphoproliferative disease**

**No**	Diagnosis*	Ohshima category**	**Histo**	**CD4**	**CD8**	**CD56**	**TCR αβ**	**TCR γδ**	**TCR PCR**	**Cell of origin**	**BM karyotype**
1	CAEBV	A1	P	-	-	-	-	-	PC	TCR silent vs NK	N
2	CAEBV	A1	P	-	+	-	+	-	PC	T	N
3	CAEBV	A1	P	-	+	+	-	-	PC	TCR silent vs NK	N
4	STLPDC	A2	P	-	+	-	+	-	MC	T	N
5	STLPDC	A2	P	-	+	-	-	-	MC	T	N
6	STLPDC	A2	P	-	+	-	-	-	MC	T	N
7	STLPDC	A2	P	+	-	+	Focal +	-	MC	T	N
8	CAEBV vs STLPDC	A1 or A2	P	NA	NA	+	NA	NA	NA	NA	NA
9	CAEBV vs STLPDC	A1 or A2	P	-	+	-	NA	NA	NA	NA	NA
10	CAEBV vs STLPDC	A1 or A2	P	+	+	-	NA	NA	NA	NA	NA
11	CAEBV vs STLPDC	A1 or A2	P	NA	NA	-	NA	NA	NA	NA	NA
12	STLPDC	A3	M	-	+	+	Focal +	-	MC	T	Abn
13	STLPDC	A3	M	-	+	-	+	-	MC	T	N
14	STLPDC	B	M	-	+	+	+	-	MC	T	Abn
15	STLPDC	B	M	-	+	-	-	-	MC	T	Abn
16	STLPDC	B	M	+	-	-	Focal +	-	PC	favour T	Abn
17	STLPDC	B	M	-	+	-	+	-	MC	T	N
18	STLPDC	B	M	-	+	-	+	-	MC	T	Mosaic
19	STLPDC	B	M	-	+	-	+	-	NA	T	N
20	STLPDC	B	M	+	-	-	+	NA	PC	T	Abn
21	STLPDC	B	M	-	-	-	NA	NA	NA^‡^	NA	NA
22	favour STLPDC	A3 or B	M	-	-	+	+	-	NA	T	NA

*Abbreviations: Histo* histology, *M* monomorphic, *P* polymorphic, *TCR* clonality for T-Cell Receptor Gamma gene by PCR, *MC* monoclonal, *PC* polyclonal, *Abn* abnormal, *N* normal, *NA* not available, *OS* overall survival, *BM* bone marrow, *CAEBV* chronic active EBV infection of T-NK type, *STLPDC* systemic EBV + T cell lymphoproliferative disorder of childhood, *EBV + TLPD* EBV-positive T cell lymphoproliferative disorder.

^‡^EBV terminal repeats analysis revealed monoclonality.

*Based on WHO classification and recommendation from NIH consensus meeting in 2008 [12].

**Based on categorization of EBV + T/NK lymphoproliferative disorders in children by Ohshima et al. [13].

Based on the WHO classification and NIH recommendation [1,12], the term ‘CAEBV’ is applied to systemic LPDs that are not frank lymphomas and that arise during primary infection and persist for over 6 months. The term ‘systemic EBV-positive T-cell LPD’, as adopted by the WHO classification, is used for those cases that are monoclonal. We also categorized the cases based on the criteria proposed by Ohshima et al. [13] (ie. morphology, clonality and clinical presentation) into the following (i) category A1, polymorphic and polyclonal LPD; (ii) category A2, polymorphic and monoclonal LPD; (iii) category A3, monomorphic and monoclonal LPD; and (iv) category B, monomorphic and monoclonal LPD with fulminant course (Table 1). We considered the presence of an abnormal karyotype and/or monoclonal TCRG gene rearrangement as indicative of a clonal proliferation [14]. Fulminant course is defined as rapid clinical progression within 1 or 2 months [2]. Follow up data was collected.

Four of the 22 cases of TNKLPD with adequate formalin fixed paraffin embedded (FFPE) tissue and good quality RNA were selected for GEP (cases 5, 7, 16, 17, see Table 2). The FFPE tissues used for GEP included lymph node and skin biopsies. FFPE control tissues from normal skin and lymph node were also included. This study is approved by the Domain Specific Review Board of the National Healthcare Group, Singapore.

**Table 2:** **Clinical, survival and treatment data of EBV + TNK-cell lymphoproliferative disease**

**No**	Ohshima category*	**Histo**	**Sex**	**Ethnicity**	**Age (yrs)**	**Tissue type**	**GEP**	**HLH**	**Treatment**	**Follow up**	**OS (mth)**
1	A1	P	M	Indonesian	3.4	BM	No	+	HLH -P	Alive	55
2	A1	P	M	Chinese	13.1	BM	No	+	HLH-P, AHCT	Dead (AHCT complications)	48
3	A1	P	M	Vietnamese	24.8	BM	No	+	Chemo	Alive	6
4	A2	P	F	Chinese	5.3	BM	No	+	HLH-P, AHCT	Alive	64
5	A2	P	M	Chinese	1.4	Skin	Yes	+	HLH-P , AHCT	Alive (graft rejection)	95
6	A2	P	F	Indonesian	6.1	BM	No	+	HLH-P	Alive	31
7	A2	P	M	Chinese	16.7	LN	Yes	-	Steroids, AHCT	Dead	14
8	A1 or A2	P	M	Japanese	4	liver	No	+	AHCT	Alive	94.4
9	A1 or A2	P	F	Japanese	19	BM	No	-	AHCT	Alive	9.2
10	A1 or A2	P	F	Japanese	19	BM	No	+	Chemo	Alive	32.5
11	A1 or A2	P	F	Japanese	39	Skin	No	-	Chemo	Dead	21.8
12	A3	M	F	Vietnamese	16.4	BM	No	+	HLH-P	Dead	0.4
13	A3	M	F	Chinese	4.8	BM	No	+	HLH-P	Alive	21
14	B	M	M	Indonesian	13.3	BM	No	+	HLH -P	Dead	0.83
15	B	M	M	Indonesian	20.9	BM	No	+	HLH -P	Dead	2
16	B	M	F	Chinese	9.5	LN	Yes	+	Supportive	Dead	1
17	B	M	M	Chinese	18.7	LN	Yes	+	HLH -P	Dead	1.16
18	B	M	M	Vietnamese	1	BM	No	+	HLH-P	NA	NA
19	B	M	M	Filipino	7.6	BM	No	+	HLH-P	Dead	1
20	B	M	F	Chinese	19.3	BM	No	+	HLH-P	Dead	0.1
21	B	M	F	Japanese	48	BM	No	+	Chemo, AHCT	Dead	2.2
22	A3 or B	M	M	Others	18.3	BM	No	NA	Supportive	Dead	0.7

*Abbreviations: M* male, *F* female, *Histo* histology, *P* polymorphic, *M* monomorphic, *HLH-P* 2004 Histiocytic Lymphohistiocytosis protocol, *AHCT* Allogeneic hematopoietic cell transplant, *+* present, *−* absent, *TCR* Clonality for TCRG gene, *R* rearranged, *NR* not rearranged, *NA* not available, *OS* overall survival, *LN* lymph node, *BM* bone marrow, *Chemo* chemotherapy. *Categorization of EBV-associated T/NK LPD in children proposed by Ohshima et al. [13].

### RNA extraction from FFPE for gene expression profiling

Total RNA from human FFPE tissues were isolated using High Pure RNA Paraffin Kit (Roche Applied Science, Mannheim Germany) according to the manufacturer’s recommendations. To achieve higher recovery of RNA, 10 μm FFPE sections were deparaffinized with 100% xylene followed by 100% ethanol. The dried tissues were then subjected to proteinase K digestion and remaining steps were performed as per the manufacturer’s protocol. Concentration and purity of total RNA samples were measured using the NanoDrop ND 3.0 spectrophotometer (NanoDrop Technologies Inc, Wilmington, DE). RNA integrity was assessed with the Agilent 2100 Bioanalyzer (Agilent Technologies, Palo Alto, CA) and the RNA 6000 LabChip kit (Agilent Technologies).

### Gene expression profiling and analysis

We conducted a genome-wide gene expression profiling (GEP) on TNKLPD and normal control FFPE samples using the Illumina WG-DASL (Whole Genome cDNA-mediated Annealing, Selection, and Ligation) (Illumina, Inc., San Diego) [16,17]. Briefly, 250 ng of total RNA from each sample were converted to biotinylated cDNA using the Illumina® TotalPrep RNA Amplification Kit (Ambion, Inc., Austin, TX, USA) according to the manufacturer’s recommendations. The cDNA targets were hybridized to HumanRef-8 Expression BeadChip arrays (Illumina, Inc., San Diego), which contain 24526 probes, for 16 hours before being washed and stained with streptavidin-Cy3 according to the manufacturer’s protocol. The beadchips were scanned and quantitated using Illumina’s Bead-Station 500GX Genetic Analysis Systems scanner. The raw signals extracted were normalized using a linear calibration method, as described in our previous paper [18]. Analysis of the data was done by R/Bioconductor.

Since we found a significantly poorer survival in patients with polymorphic infiltrate (P-group) vs patients with monomorphic infiltrate (M-group), we compared genes that are differentially expressed between P-group and M-group to identify genes that are of prognostic value. Significance analysis of microarrays (SAM) [19] was not appropriate due to the small sample size and we selected the genes with more than 4-fold change for comparison between the two groups with good and poor outcome.

### Immunohistochemistry (IHC)

In order to validate the expression of cyclin E2 and Ki67 in the CD3-positive tumor cells and not the reactive population, we performed the following double stains CD3/cyclin E2 and CD3/Ki67 on four-μm sections of TNKLPD samples [including bone marrow (BM), skin and lymph node (LN) biopsies] using the conditions listed in Additional file 2. Appropriate positive tissue controls were used. The stains were performed using Leica BondMax auto-stainer. The immunohistochemical expression for the antibodies was manually scored as a percentage of the CD3-positive tumor cell population by one of the authors (NSB), without knowledge of the clinicopathologic and GEP data. Since there is no well-established cut-off criteria for high cyclinE2 expression in the literature, we determined the median value of cyclinE2 expression in our 22 cases and defined high cyclinE2 expression as equal or greater than the median expression. The median expression of cyclinE2 was determined as 15% in our cases and high cyclinE2 expression is defined as 15% or more expression in the CD3-positive tumor cell population.

## Results

### Clinical and laboratory findings

The age of the patients ranged from 1 to 48 years old with median age at the time of diagnosis of 14.9 years (Table 2). Two of our cases occurred in patients older than 30 years of age (case 11 and 21) similar to previous observations that this disease can occasionally affect adults [4,13,14]. There were 12 males and 10 females. All the cases presented with fever. Hepatomegaly and/or splenomegaly were present in 21 of 22 patients. Nine of 18 patients with adequate data had lymphadenopathy and 21 out of 22 had cytopenias. 18 out of 21 cases fulfilled the criteria of HLH. Polymerase chain reaction (PCR) for EBV was detected in the blood of all 17 cases with adequate data.

### Pathologic features

**Figure 1:**
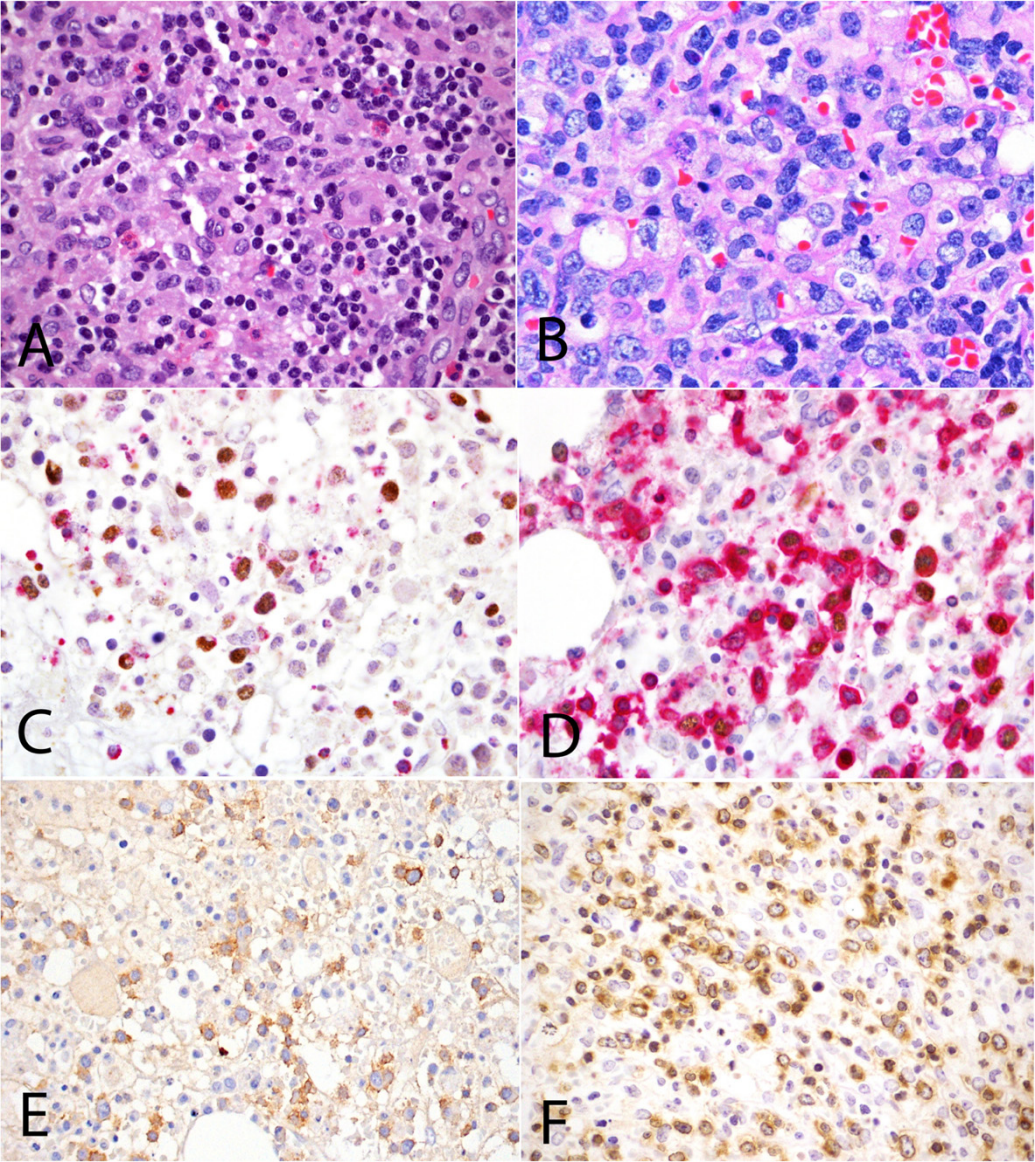
**Morphology and phenotype of TNKLPD.** Case with polymorphic infiltrate composed of mixed population of small lymphoid cells with minimal atypia, histiocytes and eosinophils (**A**, H&E, original magnification 600×). Monomorphic infiltrate consisting of large malignant lymphoid cells with irregular nuclei (**B**, H&E, original magnification 600×). Expression of cytotoxic marker TIA1 in EBER-positive tumour cells (**C**, EBER/TIA1 double stain, EBER stains nucleus brown and TIA1 stains cytoplasmic granules red, original magnification 1000×). Positive expression for EBER in situ hybridization in CD3-positive tumor cells (**D**, EBER/CD3 double stain, EBER stains nucleus brown and CD3 stains cell membrane/cytoplasm red, original magnification 1000×). Positive expression for CD56 (**E**, CD56, original magnification 600×) and TCRbeta (**F**, TCRB, original magnification 600×). All photographs were taken with a DP20 Olympus camera (Olympus, Tokyo, Japan) using an Olympus BX41 microscope (Olympus). Images were acquired using DP Controller 2002 (Olympus) and processed using Adobe Photoshop version 5.5 (Adobe Systems, San Jose, CA, USA).

Microscopically, the biopsies from BM, LN and skin showed a broad spectrum of appearance ranging from polymorphic cellular infiltrate (Figure 1A) composed of abundant reactive cells, to overtly malignant monomorphous lymphoid proliferation (Figure 1B). Hemophagocytosis is evident in the majority of cases. Eleven of 22 cases revealed polymorphic cellular infiltrate while the rest showed monomorphic lymphoid infiltrate (Table 1).

Phenotypically, all cases revealed a CD3+ cytotoxic T/NK-cell lymphoid infiltrate which expresses EBER by in-situ hybridization (Figures 1C and D). Of the 20 cases tested, 13 were CD4-/CD8+ and 3 were CD4-/CD8-. Another three were CD4+/CD8- and one case was CD4+/CD8+. Expression of CD56 was present in 6 cases (Figure 1E). TCRαβ immunoreactivity was seen in 12 of 17 cases (Figure 1F) tested. All cases studied were negative for TCRγδ protein. Monoclonal T-cell receptor gamma gene rearrangement was detected in 10 out of 15 patients, of which 4 cases revealed a polymorphic proliferation. Bone marrow cytogenetics was performed in 16 cases, of which 5 revealed abnormal karyotypes.

With regards to cell lineage, 15 of the 17 cases (88%) with adequate workup were of T cell lineage; 10 of these 15 cases had clonal rearrangement of TCRG gene by PCR, the remaining 5 cases showed positive expression for TCRαβ. Two cases (1 and 3) were negative for TCRαβ and TCRγδ, and TCRG gene was polyclonal. These 2 cases may represent either a TCR-silent or NK phenotype (Table 1).

### Categorization of TNKLPD, treatment and clinical outcome

The diagnosis of the cases based on WHO and NIH proposed nomenclature [12] as well as the corresponding categorization as proposed by Ohshima et al. [13] are provided in Table 1. Three cases were classified as CAEBV and 15 were STLPDC. The remaining 4 cases were difficult to classify accurately because of lack of clonality data. All the CAEBV cases had polymorphic infiltrate and are polyclonal by definition (Ohshima type A1). Of the 15 STLPDC, 4 cases had polymorphic and monoclonal infiltrate, corresponding to Ohshima type A2. The remaining 11 STLPDC cases showed monomorphic proliferation. Case 12 was categorised as Ohshima type A3 although the patient died 0.4 month after diagnosis because she presented with symptoms for 2 years prior to diagnosis. There were no cases of aggressive NK cell leukemia and all our cases with aggressive clinical course were of T cell origin.

Follow up was available in 21 cases with median of 11.6 months (ranged from 3 days to 8.9 years). The overall median survival was 21.8 months. Of the 18 cases that could be classified, the median survival for STLPDC was 2 months compared to median survival not yet reached for CAEBV. However, this difference was not significant and may be related to small sample size (p = 0.0668).

**Figure 2:**
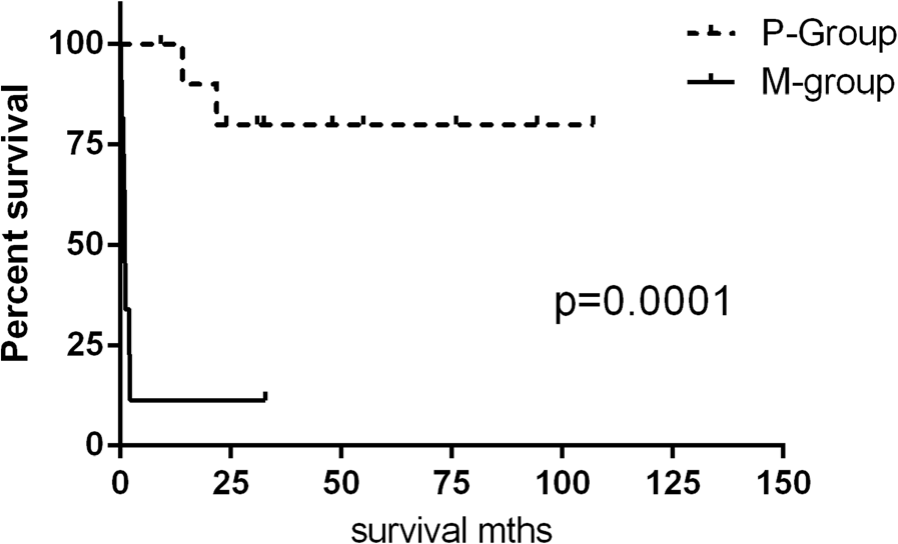
**Statistically significant difference in survival between P-group (A1 and A2 subtypes) and -M-group (A3 and B subtypes) cases.**

Interestingly, cases with polymorphic infiltrate (P-group) and those with monomorphic infiltrate (M-group) showed a statistically significant difference in survival with median survival not yet reached in P-group and 1 month in M-group (p = 0.0001) (Figure 2). We did not find any significant difference in survival between cases with polyclonal and monoclonal lymphoid infiltrate, although this may again be due to small sample size.

Five of 11 patients in P-group were treated with 2004 HLH protocol, a chemo-immunotherapy which includes etoposide, dexamethasone, cyclosporine A upfront and, in selected patients, intrathecal therapy with methotrexate and corticosteroids [15] (Table 2). Six P-group cases also received allogeneic hematopoietic cell transplant (AHCT), of which one died of disease and one died from AHCT related complications. The other 4 patients who received AHCT remained alive although one patient had graft rejection. Overall, 8 out of 11 P-group patients remained alive. In contrast, 9 of 10 patients in M-group with follow up data died of disease and only one patient remained alive at 21 months of follow up. Eight of 11 M-group patients were treated with 2004 HLH protocol while the other 2 received supportive management. Except for one M-group case who received chemotherapy and AHCT, none of the other cases received AHCT because of the rapid clinical progression.

### Cyclin E2 expression is associated with high proliferation and poor outcome in TNKLPD

**Figure 3:**
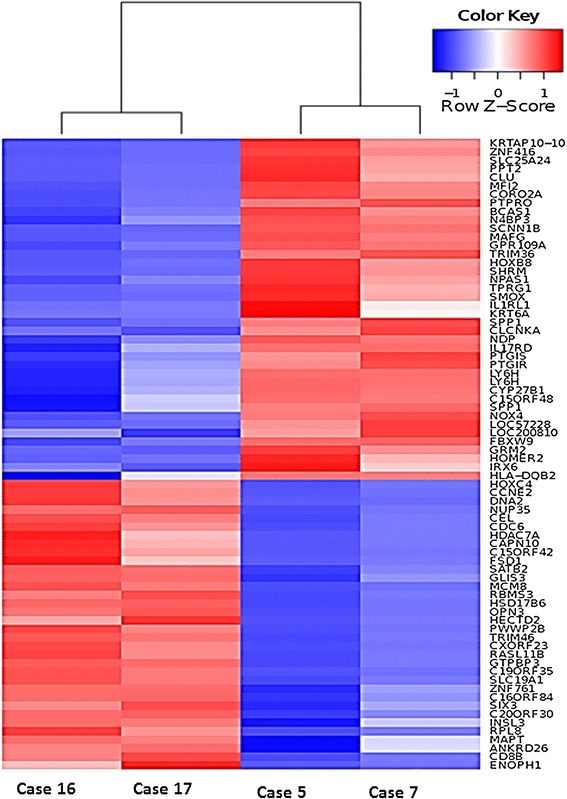
**Differentially expressed genes between P-group and M-group patients with TNKLPD.**

Of the 4 cases that were profiled, 2 cases belonged to P-group (polymorphic infiltrate) and the other 2 were in M-group (monomorphic infiltrate). Since the 2 groups showed significant difference in survival, we compared the genes differentially expressed between the two groups in order to identify targets that are associated with poor outcome. A total of 74 genes showed a 4-fold difference in expression between the 2 groups, of which 34 were upregulated (including CCNE2/cyclin E2) and 40 were downregulated in the group with shorter survival (Figure 3).

**Figure 4:**
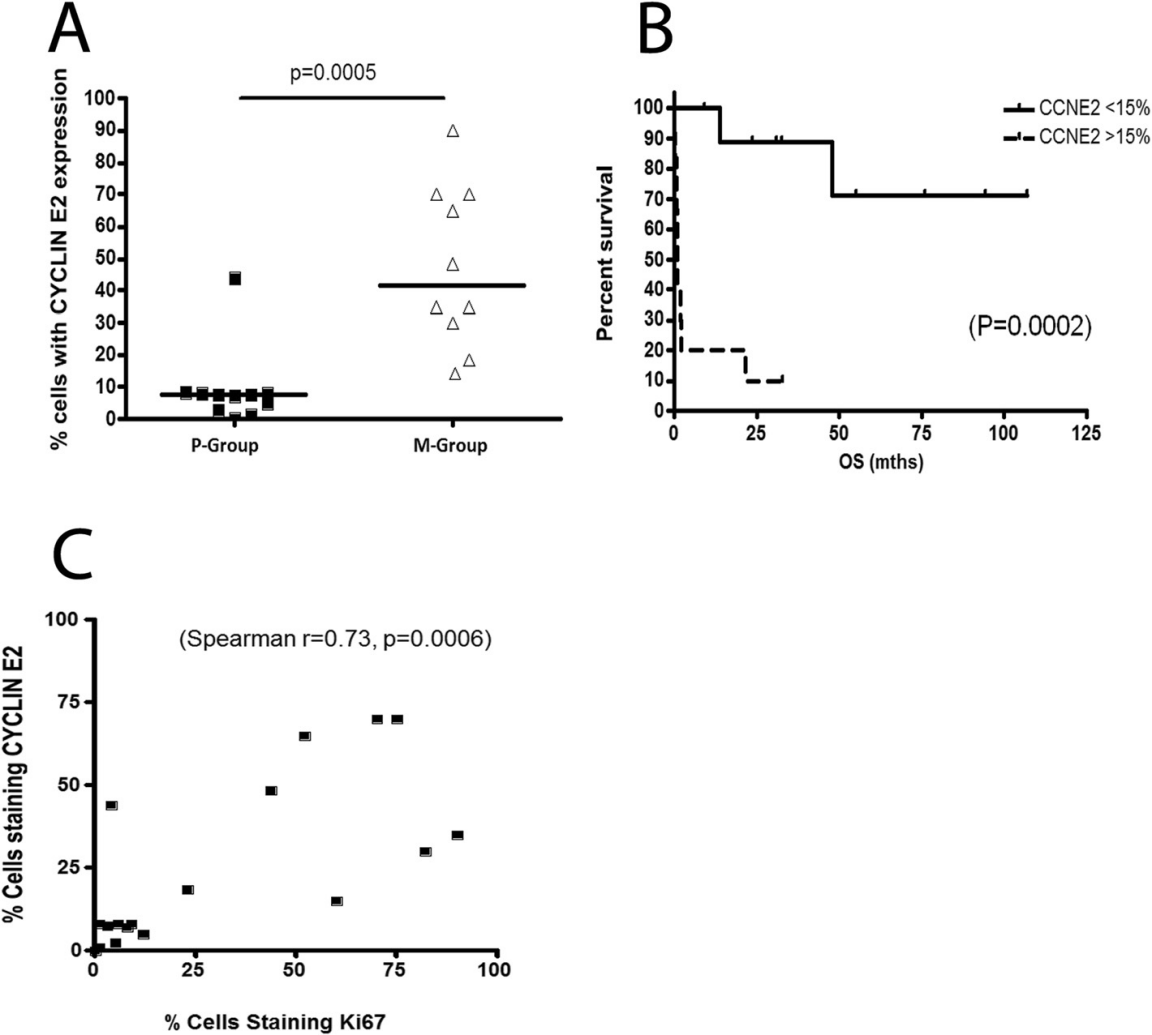
**Cyclin E2 expression was associated with high proliferation rate and poor outcome in TNKLPD.** Significantly higher cyclin E2 expression was present in M-group compared to P-group patients (**A**, median expression of 42% compared with 8%, p = 0.0005). Patients with high tumor cyclin E2 expression of 15% or greater had statistically significant shorter survival compared with those with low cyclin E2 expression less than 15% (**B**, p = 0.0002). High cyclin E2 expression is highly correlated with high Ki-67 proliferation rate (**C**, spearman r = 0.73, p = 0.0006).

**Figure 5:**
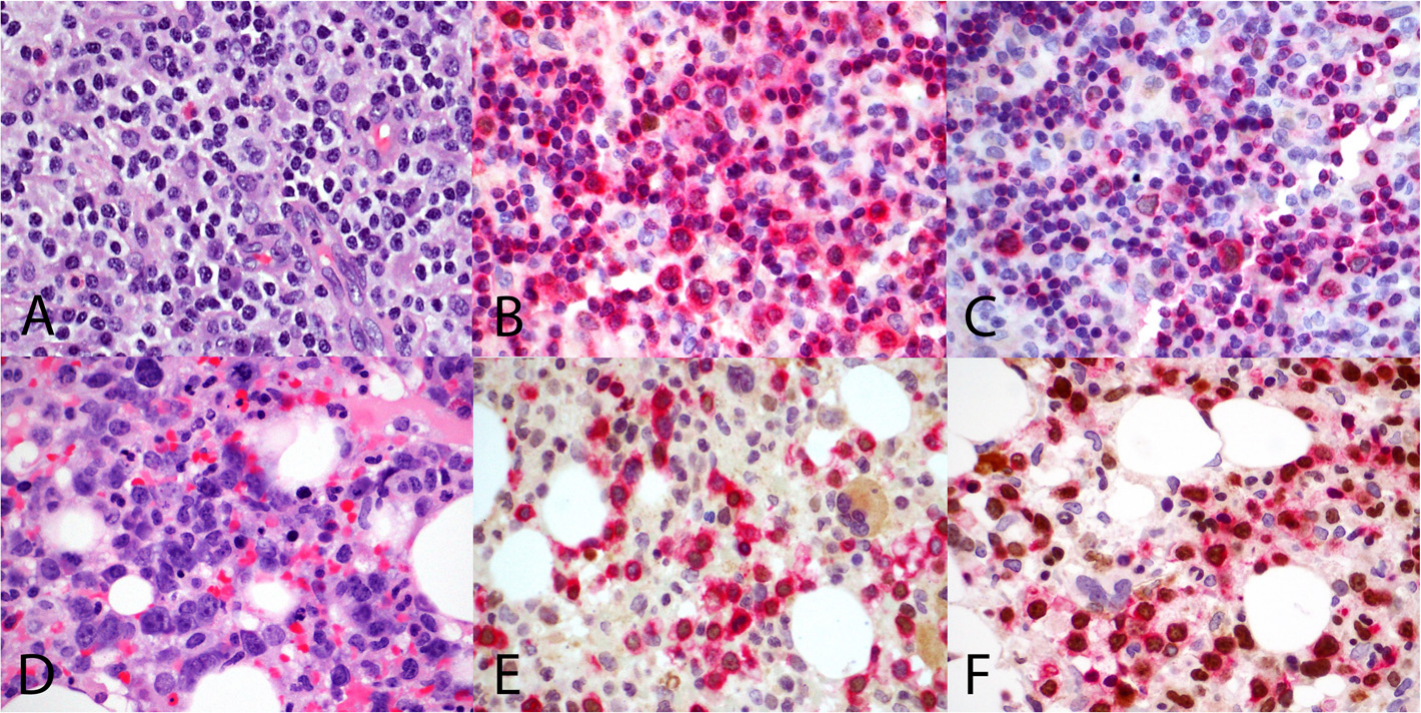
**Immunohistochemical expression of cyclin E2 protein and Ki67 proliferation rate in M-group compared to P-group cases.** Case 7 with type A2 disease and polymorphic morphology (P-group) (**A**, H&E original magnification 600×) showing low cyclin E2 expression (**B**, CD3/cyclin E2 double stain, original magnification 600×) and low Ki67 proliferation (**C**, CD3/Ki67 double stain, original magnification 600×). Case 19 with type B disease and monomorphic morphology (M-group) (**D**, H&E original magnification 600×) with moderately high cyclin E2 expression (**E**, CD3/cyclin E2 double stain, original magnification 600×) and high Ki67 proliferation (**F**, CD3/Ki67 double stain). CD3 stains cell membrane/cytoplasm red, and cyclin E2 and Ki67 stain nucleus brown. All photographs were taken with a DP20 Olympus camera (Olympus, Tokyo, Japan) using an Olympus BX41 microscope (Olympus). Images were acquired using DP Controller 2002 (Olympus) and processed using Adobe Photoshop version 5.5 (Adobe Systems, San Jose, CA, USA).

In order to validate our GEP findings, we performed immunohistochemistry on a few selected genes of interest and we found that there is a significant overexpression of cyclin E2 protein in M-group patients compared to P-group (median expression of 42% compared with 8%, p = 0.0005) (Figure 4A, Table 3). There is also a statistically significant difference in survival between patients with low vs high cyclin E2 expression (median survival not yet reached vs 1.16 months, p = 0.0002) (Figure 4B) suggesting a potential role of cyclin E2 as an adverse prognostic marker in TNKLPD. Interestingly, only one case in P-group (case 11) had a high cyclin E2 expression and this patient also died of disease less than 2 years after diagnosis. Since cyclin E2 has an important role in cell cycle progression, we also performed Ki-67/CD3 double stain to determine if the upregulation of cyclin E2 in M-group of TNKLPD correlated with a higher proliferation rate in this group. Indeed, we found a significant correlation between cyclin E2 and Ki-67 with spearman correlation r = 0.73, p = 0.0006 (Figure 4C, Table 3, Figure 5). The IHC of other selected genes were not included because of technical difficulty in performing double stains in TNKLPD tissue samples which included mostly bone marrow biopsy sections.

**Table 3:** **Immunohistochemical expression of cyclin E2 and Ki67 in CD3-positive tumor population of TNKLPD**

**Case No.**	**Diagnosis**	**Ohshima category**	**Histology**	**Cyclin E2 (%)**	**Ki67 (%)**	**Follow up**	**OS (mth)**
1	CAEBV	A1	P	3	5	Alive	55
2	CAEBV	A1	P	8	NA	Dead (AHCT complications)	48
3	CAEBV	A1	P	0	0	Alive	6
4	STLPDC	A2	P	8	6	Alive	64
5	STLPDC	A2	P	1	1	Alive cgraft rejection)	95
6	STLPDC	A2	P	5	12	Alive	31
7	STLPDC	A2	P	8	3	Dead	14
8	CAEBV vs STLPDC	A1 or A2	P	8	1	Alive	94.4
9	CAEBV vs STLPDC	A1 or A2	P	7	8	Alive	9.2
10	CAEBV vs STLPDC	A1 or A2	P	8	9	Alive	32.5
11	CAEBV vs STLPDC	A1 or A2	P	44	4	Dead	21.8
12	STLPDC	A3	M	90	NA	Dead	0.4
13	STLPDC	A3	M	70	75	Alive	21
14	STLPDC	B	M	49	44	Dead	0.83
15	STLPDC	B	M	35	NA	Dead	2
16	STLPDC	B	M	70	70	Dead	1
17	STLPDC	B	M	15	60	Dead	1.16
18	STLPDC	B	M	30	82	NA	NA
19	STLPDC	B	M	35	90	Dead	1
20	STLPDC	B	M	18	23	Dead	0.1
21	STLPDC	B	M	65	52	Dead	2.2
22	favour STLPDC	A3 or B	M	NA	NA	Dead	0.7

*Abbreviations: NA* not available, *mth* months, *P* polymorphic, *M* monomorphic.

## Discussion

Chronic active EBV infection of T/NK type (CAEBV-T/NK), aggressive NK cell leukemia (ANKL) in children and systemic EBV + T-cell lymphoproliferative disorders (LPD) of childhood are a group of systemic EBV-associated T/NK cell LPD that is prevalent in children and young adults in Asia, Native Americans in Mexico and South America [12,20]. Although rare, this is an important group of disease to study because very little is known about the disease pathogenesis, and they are often aggressive in behavior with no effective treatment. Furthermore, biologic markers useful in predicting patient outcome have also not been identified. This is attributed to the rarity of the disease and limited availability of tissue because most of the tissue samples from such patients are bone marrow biopsies containing scanty amount of tumor of suboptimal quality secondary to decalcification that is required for the processing of bone marrow tissue samples.

In this study, we describe 22 cases of TNKLPD in Singapore and Japan which includes CAEBV-T/NK and systemic EBV + T-cell LPD in childhood. Although TNKLPD affects mainly children and young adults, two of our cases occurred in patients older than 30 years, similar to observations from previous studies that this disease can occasionally occur in older adults [4,13,14]. There were no aggressive NK cell leukemia (ANKL) and all our TNKLPD with fulminant clinical course (Ohshima B subtype) were of T cell origin and classified as systemic EBV-positive T cell LPD of childhood. The lack of systemic fulminant TNKLPD disease of NK origin may be due to more comprehensive testing for TCR clonality in combination with immunohistochemistry for the expression of TCRαβ and TCRγδ. Since antibodies for TCRαβ and TCRγδ for formalin fixed paraffin sections are only available in the recent years, it is possible that some of the ANKL diagnosed in the past may actually be of T cell origin due to limited T cell assessment.

We found that cases with monomorphic infiltrate (M-group) had significantly shorter survival compared to P-group cases with polymorphic infiltrate. In our study, all but one case, with a monomorphic infiltrate had an overall survival of less than 3 months. This highlights the importance of morphology in predicting outcome and that cases with frank malignant proliferation should be regarded as an aggressive lymphoma and treated as such. This is in line with the recommendation from the consensus meeting in NIH 2008 that the term “CAEBV” should not be applied to systemic LPDs that contain a frankly malignant infiltrate, ie. Ohshima A3 and B subtypes [12].

Recently, Quintanilla-Martinez et al. described 20 cases of Hydroa vacciniforme–like lymphoma (HVLL), which is also an EBV-positive T-cell lymphoproliferative disorder of childhood but with predominant cutaneous manifestations [21]. The authors found that although HVLL in general showed a favorable response to conservative therapy, there is a risk to develop systemic lymphoma and criteria such as presence of systemic symptoms, T-cell clonality, amount of EBV-positive cells, and/or density of the infiltrate do not help in predicting which patients will eventually progress to systemic disease [21]. Hence, the challenge remains to identify morphological or clinical markers to predict outcome or clinical progression. Importantly, in this study of EBV-associated T/NK LPD with primarily systemic presentation, our GEP data revealed cyclinE2 gene to be overexpressed in M-group compared to P-group patients. Using immunohistochemistry double stains, we found a corresponding higher protein expression of cyclin E2 in M-group patients and this is associated with poor outcome, thus supporting the validity of our GEP data. We further showed that the upregulation of cyclinE2 in patients with poor outcome is significantly correlated with a higher Ki67 proliferation rate.

The question of whether cyclin E2 is merely a link in the chain of events that lead to cell proliferation or whether it is a driving force in cell replication in TNKLPD is difficult to ascertain at present. In some neoplasms, cyclin E gene amplification and protein accumulation are late events [22,23] whereas in other tumors an increase in cyclin E is observed early in the progression to malignancy [24,25]. Since cyclinE2 overexpression is associated with high Ki67 proliferation, monomorphic large cell morphology and poor outcome, it is tempting to speculate that the upregulation of cyclinE2 is merely a reflection of tumor progression in TNKLPD. However, one patient (case 11) in this study showed polymorphic morphology and low Ki67 proliferation, but high cyclinE2 expression. This patient had a fairly aggressive outcome and died of disease within 2 years of diagnosis, suggesting that cyclinE2 upregulation in some TNKLPD may not simply be due to proliferation and disease progression. It is also interesting to note that, unlike cyclin E1 and Ki67 which are expressed in most proliferating normal and tumor cells, cyclin E2 levels are often low to undetectable in non-transformed cells and increased significantly in tumor-derived cells [26]. Hence, it is possible that cycinE2 may be a more specific marker of tumor proliferation than Ki67.

## Conclusion

We described 22 cases of TNKLPD with systemic presentation, including CAEBV of T/NK type and systemic EBV + T cell LPD of childhood and found the presence of a monomorphic infiltrate and high cyclin E2 and Ki-67 expression to be associated with poor outcome in TNKLPD, which is a rare disease with no known markers of prognostic significance to date. Whether cyclin E2 alone or in combination with Ki67 and morphology allows better prognostic stratification than standard morphology requires further study with larger sample size.

## Additional files

Additional file 1:
**Comparison of the proposed nomenclature of EBV-positive T/NK lymphoproliferative disorder (LPD) with systemic presentation.**
Additional file 2:
**Summary of immunohistochemical double stain conditions.**
