# Supplementary material for: Prognostic implication of morphology, cyclinE2 and proliferation in EBV-associated T/NK lymphoproliferative disease in non-immunocompromised hosts
Source: Orphanet J Rare Dis. 2014 Dec 5;9:165. doi: 10.1186/s13023-014-0165-x (PMC4263108; doi:10.1186/s13023-014-0165-x)
Supplement: Additional file 1: — Comparison of the proposed nomenclature of EBV-positive T/NK lymphoproliferative disorder (LPD) with systemic presentation. [file 13023_2014_165_MOESM1_ESM.doc]

Additional file 1

Comparison of the proposed nomenclature of EBV-positive T/NK lymphoproliferative disorder (LPD) with systemic presentation.

| **Proposed nomenclature according to the 4^th^ ASIAN Hematopathology Workshop*** | **Corresponding category by Ohshima et al**** | **Consensus meeting NIH/WHO 2008***** |
| --- | --- | --- |
| 1. Systemic EBV-positive T/NK cell LPD of childhood type^a^ | B | Systemic EBV+ T cell LPD of childhood |
| 2. Chronic active EBV disease-type T/NK cell LPD |  |  |
| i. Polymorphic/polyclonal | A1 | CAEBV-T/NK |
| ii. Polymorphic/monoclonal | A2 | Systemic EBV+ T cell LPD of childhood |
| iii.Monomorphic/monoclonal | A3 | Systemic EBV+ T cell LPD of childhood |

Abbreviations: CAEBV, chronic active EBV infection; LPD, lymphoproliferative disorder;

^a^Includes aggressive NK cell leukemia in children. The proliferating cells are polyclonal, oligoclonal, or monoclonal T/NK cells

* Consensus report of the 4th Asian Hematopathology Workshop on EBV-associated T and NK cell lymphoproliferative disorders.

** Categorization of EBV-associated T/NK LPD in children proposed by Ohshima et al.

*** Status report and summary of an international meeting on Epstein-Barr virus-associated lymphoproliferative disease in non-immunocompromised hosts.

References

1. Ko Y-H, Kim H-Y, Oh Y-H, Park G, Lee S-S, Huh J, Kim C-W, Kim I, Ng S-B, Tan S-Y, Chuang S-S, Nakamura N, Yoshino T, Nagamura S, Kimura H, Ohshima K: **EBV-associated T and NK cell lymphoproliferative disorders: consensus report of the 4th Asian Hematopathology Workshop.** *J Hematopathol* 2012, **5:**319-324.

2. Ohshima K, Kimura H, Yoshino T, Kim CW, Ko YH, Lee SS, Peh SC, Chan JK: **Proposed categorization of pathological states of EBV-associated T/natural killer-cell lymphoproliferative disorder (LPD) in children and young adults: overlap with chronic active EBV infection and infantile fulminant EBV T-LPD.** *Pathol Int* 2008, **58:**209-217.

3. Cohen JI, Kimura H, Nakamura S, Ko YH, Jaffe ES: **Epstein-Barr virus-associated lymphoproliferative disease in non-immunocompromised hosts: a status report and summary of an international meeting, 8-9 September 2008.** *Ann Oncol* 2009, **20:**1472-1482.
