# Supplementary material for: Prognostic implication of morphology, cyclinE2 and proliferation in EBV-associated T/NK lymphoproliferative disease in non-immunocompromised hosts
Source: Orphanet J Rare Dis. 2014 Dec 5;9:165. doi: 10.1186/s13023-014-0165-x (PMC4263108; doi:10.1186/s13023-014-0165-x)
Supplement: Additional file 2: — Summary of immunohistochemical double stain conditions. [file 13023_2014_165_MOESM2_ESM.docx]

Additional file 2

Summary of immunohistochemical double stain conditions

| **Antibody** | **Source** | **Clone** | **Dilution** | **Retrieval** | **Incubation** | **Chromogen** |
| --- | --- | --- | --- | --- | --- | --- |
| CyclinE2/CD3 | Cyclin E2: Epitomic | EP454Y | 1:50 | ER2-30' | 60' | DAB |
|  | CD3: DAKO | Polyclonal | 1:25 to 1:50 | – | 15' | AP |
| Ki67/CD3 | Ki67: DAKO | MIB1 | 1:100 to 1:200 | ER2-20’ | 15’ | DAB |
|  | CD3: DAKO | Polyclonal | 1:25 to 1:50 | - | 15’ | AP |

Abbreviations: DAB, diaminobenzidine (brown stain); AP, alkaline phosphatise (red stain)
